# T_1_ Relaxation Time for the Prediction of Renal Transplant Dysfunction

**DOI:** 10.3389/ti.2025.14301

**Published:** 2025-04-10

**Authors:** Haris Omić, Farsad Eskandary, Dietrich Beitzke, Marcos Wolf, Nicolas Kozakowski, Georg Böhmig, Andrea Beck-Tölly, Michael Eder

**Affiliations:** ^1^ Division of Nephrology and Dialysis, Department of Medicine III, Medical University of Vienna, Vienna, Austria; ^2^ Division of Cardiovascular and Interventional Radiology, Department of Biomedical Imaging and Image-Guided Therapy, Medical University of Vienna, Vienna, Austria; ^3^ Center for Medical Physics and Biomedical Engineering, Medical University of Vienna, Vienna, Austria; ^4^ Department of Pathology, Medical University of Vienna, Vienna, Austria; ^5^ Division of General and Paediatric Radiology, Department of Biomedical Imaging and Image-Guided Therapy, Medical University of Vienna, Vienna, Austria

**Keywords:** kidney transplantation, T1 relaxation time, allograft dysfunction, non-invasive, biomarkers

## Abstract

Quantitative magnetic resonance imaging (MRI) is emerging as a non-invasive tool to measure tissue scarring in renal allografts. However, whether prolonged T_1_ relaxation time results in lower transplant survival rates is unknown. This retrospective cohort study analyzed the capability to predict renal allograft dysfunction based on median T_1_ time. Forty-six transplant recipients with non-contrast 1.5T MRI and allograft biopsy were included. The primary endpoint was the eGFR slope over 24 months. T_1_ relaxation time correlated significantly with eGFR levels at all follow-up stages. Patients with T_1_ relaxation time above the median (T_1_^high^) had a consistent decline in kidney function as compared to the patient group below the median (T_1_^low^): overall eGFR slope: 11.3 vs. 1.4 mL/min/1.73 m^2^ over 24 months, p = 0.016. Graft survival rates at 24 months were 52% in the T_1_^high^ vs. 87% in the T_1_^low^ group, p = 0.0015. ROC analysis discovered a positive predictive value of 52% and a negative predictive value of 91% for graft loss. T_1_ mapping identified patients with a persistent decline of allograft function and an increased risk of allograft loss. MRI could significantly influence monitoring strategies in transplant surveillance, offering a safe, non-invasive alternative to traditional diagnostic methods.

## Graphical Abstract

**Figure:**
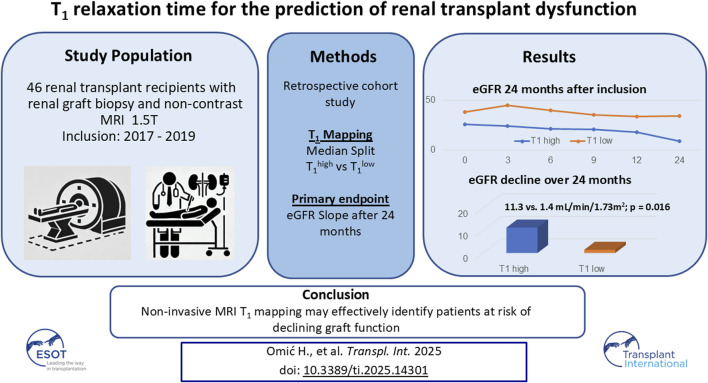

## Introduction

Kidney transplantation is the preferred treatment for end-stage renal disease [1]. One cornerstone of mitigating renal allograft dysfunction lies in the early, accurate diagnosis of graft pathologies and prompt initiation of treatment. Ideally, a diagnostic tool should detect allograft dysfunction, differentiate between its etiologies, and monitor graft function throughout therapeutic interventions, all while minimizing patient risk.

Currently, percutaneous biopsies are the gold standard for diagnosing kidney allograft pathologies. However, the procedure is not without risks, including a significant complication rate of up to 2% in transplanted kidneys [2, 3]. Biopsies are also susceptible to interobserver variability and sampling errors, which can compromise diagnostic accuracy [4, 5]. Furthermore, practical limitations such as anticoagulation therapy, hypertension, urinary infections, or simply the patient’s subjective refusal may delay a biopsy and, consequently treatment initiation. Especially in the field of renal transplantation, where sequential biopsies are common, there is an emerging interest in exploring the potential of magnetic resonance imaging (MRI) as a complementary non-invasive diagnostic tool [6–11]. MRI is distinguished by its exceptional soft tissue contrast. Its evolution, particularly in enhancing temporal and spatial resolution, has broadened its application and allows assessing functional aspects of the kidney, including renal perfusion and tissue oxygenation [12–15].

In a recent study from our center, we demonstrated a significant correlation between advanced interstitial fibrosis (Banff ci) and high cortical T_1_ [8]. T_1_ was also significantly associated with other chronic lesion markers such as tubular atrophy (Banff ct), glomerular basement membrane double contours (Banff cg), and vascular intimal thickening (Banff cv). This implies that histological scarring leads to local microstructural magneto-chemical alterations, quantifiable by MRI [15, 16]. Similar findings were also reported by other studies exploring the relationship between apparent diffusion coefficient (ADC), T_1_ and T_2_ in various kidney allograft pathologies [17–19].

However, previous publications mostly focused on correlations between MRI and biopsy findings measured at one-time point cross-sectionally. The longitudinal assessment of allograft function in relation to T_1_ values was studied to a much smaller extent. Due to less risk of sampling error in MRI assessments, it may be hypothesized that T_1_ mapping could even exceed the prognostic value of histologically-quantified lesion markers.

A study from Berchtold et al. showed that ADC was able to predict the progression of interstitial fibrosis more reliably than serum creatinine alone [20]. Yet, to our knowledge, it is unexplored whether high T_1_ subsequently precedes reduced allograft survival. To test this hypothesis, we analyzed the course of graft function in a group of 46 patients who underwent transplant biopsies and cortical T_1_ mapping.

## Materials and Methods

### Study Design and Patient Cohort

The aim of this retrospective cohort study was to analyze the course of renal allograft function in a group of 46 transplant recipients who underwent both MRI and transplant biopsy simultaneously. Thirty-two of those patients were included in our previous prospective study, which focused on assessing correlations between T_1_ mapping, Banff lesion scores, and conventional graft function parameters [8]. The other fourteen patients underwent MRI before the initial study due to clinical indications and as part of a quality assurance protocol to test its basic feasibility.

Patients were screened for study inclusion at our outpatient clinic. Detailed inclusion criteria are provided in the study from Beck-Tölly et al. [8]. All suitable renal transplant patients scheduled for protocol or indication biopsies were actively asked for study participation. The main inclusion criteria were: age over 18 years and an estimated glomerular filtration rate (eGFR) of more than 10 mL/min/1.73 m^2^ (calculated using the Modification of Diet in Renal Disease formula). Exclusion criteria included MRI-incompatible metallic implants or pacemakers, claustrophobia, and pregnancy. Recruitment took place from December 2017 to January 2019. Non-contrast MRI scans were performed shortly before or after the biopsy, using a whole-body 1.5 T MR system (MAGNETOM Avanto Fit; Siemens Healthineers; Erlangen, Germany).

The primary endpoint was the course of graft function after assessment of baseline MRI T_1_. Longitudinal graft function was calculated based on serum creatinine levels measured in a three-month interval over the period of 24 months after the MRI. To further quantify changes in kidney function, the eGFR delta (ΔeGFR) and eGFR slopes were calculated for each observation period.

The secondary endpoint was the frequency of death-censored graft loss in relation to baseline T_1_. Graft loss was defined as the resumption of dialysis. All participants provided informed consent. Ethical approval for the study was granted by the institutional ethics committee (Approval No. 1893/2017). The study adhered to Good Clinical Practice guidelines, the principles of the Declaration of Helsinki, and the Declaration of Istanbul.

### MRI

MRI protocols and methods used in this study have been described in detail elsewhere [8]. In short, we extracted T_1_ measurements from our multiparametric MRI images, measured across three paraxial (cranial, middle, caudal) and three paracoronal (anterior, middle, posterior) planes, involving six independent regions of interest per plane. The median of those 36 measurements was defined as the overall median T_1_ cortical relaxation time. The choice to focus this current analysis on T_1_ was based on results from preceding research, which estimated kidney function based on T_1_ in patients with glomerulonephritis [21], as well as one study quantitatively evaluating renal function and renal fibrosis in patients with chronic kidney disease [22].

### Biopsy

Morphologic lesions were assessed on formalin-fixed paraffin-embedded sections using standard methodology [8]. Banff single lesions and rejection phenotypes were scored based on the Banff 2017 scheme [23]. In addition to Banff criteria, chronic structural damage in kidney grafts was assessed using the chronicity index as described by Haas et al. [24]. This index combines four key histological features: interstitial fibrosis (ci), tubular atrophy (ct), vascular fibrous intimal thickening (cv), and chronic glomerulopathy (cg). Each feature was scored on a scale from 0 (no changes) to 3 (severe changes), with the chronic glomerulopathy score being doubled. The total chronicity index ranged from 0 to 15, with higher scores indicating more significant chronic injury.

### Statistical Analysis

Continuous variables were reported as means with standard deviations (SD) or medians with interquartile ranges (IQR). Categorical variables were summarized as counts and percentages. The median split method was employed to divide patients into two groups of equal size based on the overall T_1_. Hence, the “T_1_^high^” group referred to patients with T_1_ values above and the “T_1_^low^” group for patients with T_1_ values below the median. Spearman´s correlation coefficients were used to analyze the associations between T_1_ and baseline variables, including transplant age, baseline eGFR, and the histological parameters ci, ct—as well as the chronicity index. To compare the predictive validity of Banff ci scores with T_1_, Fisher’s Z transformation was performed.

The linear mixed-effects model was performed to analyze the changes in the estimated glomerular filtration rate (eGFR slope) over time between the groups.

To compare graft survival, the Kaplan-Meier survival curve and log-rank test were calculated. To address the loss of graft function and the subsequent missing data points in our longitudinal follow-up, we implemented the “last observation carried forward” (LOCF) imputation method. Additionally, the Receiver Operating Characteristic (ROC) analysis was performed to evaluate the ability of T_1_ to predict the occurrence of allograft loss. The p-value of <0.05 was considered statistically significant.

Statistical computations and analyses were conducted using SPSS for Mac Version 20 (SPSS Inc., Chicago, IL), GraphPad Prism (GraphPad Prism 10.0.3 (217) Macintosh Version by Software MacKiev ^©^ 1994–2023 GraphPad Software, LLC), R (R Core Team, 2023) and RStudio (2022 by Posit Software, PBC).

## Results

### Study Population

Forty-six patients were included, 30 (65%) were male; the mean age at transplantation was 54.3 ± 14.8 years (mean ± SD). Baseline parameters of the total group and the subgroups (T_1_^high^ and T_1_^low^) are displayed in Table 1. The majority of patients (80.4%) received deceased donor kidneys. The median time from transplantation to study inclusion was 3 years (IQR 0.7–11.2). Six (13%) participants underwent magnetic resonance imaging before [4 ± 2.5 days, (mean ± SD)] and 38 (82.6%) after (7.9 ± 9 days) the biopsy. Two (4.4%) patients had the MRI on the day of the biopsy. The median cortical T1 was 1,369 ms (IQR 1,279–1,511). The median eGFR at the time of biopsy was 30.8 mL/min/1.73 m^2^ (IQR 20.1–49.6). Fourteen (30.4%) patients reached the endpoint graft loss. Four patients (8.6%) were lost to follow-up before the end of our observation period of 24 months.

**TABLE 1:** Baseline parameters of the study population.

Variable	Total n = 46	T_1_^high^ n = 23	T_1_^low^ n = 23	P-value
Male sex, n (%)	30 (65.2)	21 (91.3)	9 (56.2)	**<0.01**
BMI, mean ± SD	25.5 ± 3.7	25.8 ± 3.9	25.3 ± 3.7	0.72
Recipient age (years), mean ± SD	54.3 ± 14.8	54.2 ± 17.3	54.4 ± 12.3	0.95
Deceased donor, n (%)	37 (80.4)	19 (82.6)	18 (78.3)	0.50
First transplantation, n (%)	34 (73.9)	18 (78.2)	16 (69.6)	0.43
Biopsy after Tx (years), median (IQR)	3 (0.7 to 11.2)	3 (1 to 12)	1 (0 to 9)	0.26
Protocol biopsy n (%)	9 (19.6)	1 (4.3)	8 (34.8)	0.02
HLA mismatch, median (IQR)	3 (2 to 4)	3 (2 to 4)	2 (2 to 3)	0.21
Rejection diagnosed in biopsy, n (%)	14 (30.1)	7 (30.4)	7 (30.4)	>0.99
AMR	9 (19.6)	6 (26.1)	3 (13.0)	0.45
TCMR	5 (10.9)	1 (4.3)	4 (17.4)	0.34
Borderline TCMR	3 (6.5)	0 (0.0)	3 (13.0)	0.23
Banff1A	1 (2.2)	0 (0.0)	1 (4.3)	>0.99
Banff2A	1 (2.2)	1 (4.3)	0 (0.0)	>0.99
BKPyVAN	3 (6.5)	0 (0.0)	3 (13.0)	0.23
TMA	1 (2.2)	1 (4.3)	0 (0.0)	>0.99
eGFR 3 m before biopsy, (mL/min/1.73 m^2^), median (IQR)	31.7 (22.1 to 54.0)	28.6 (22.1 to 60.8)	34.9 (22.6 to 50.3)	0.92
eGFR 1 m before biopsy, (mL/min/1.73 m^2^), median (IQR)	32.3 (23.5 to 49.0)	27.3 (17.9 to 43.6)	42.0 (26.9 to 51.9)	0.08
eGFR at biopsy, (mL/min/1.73 m^2^), median (IQR)	30.8 (20.1 to 49.6)	25.56 (19.5 to 43.3)	37.9 (22.1 to 53.2)	0.20
Proteinuria (mg/g), median (IQR)	484.5 (130.5 to 1,750.25)	1717 (365 to 2,914)	193 (101 to 665)	**<0.01**
Albuminuria (mg/g), median (IQR)	209 (32.5 to 1,256.5)	1,200 (164–2,710)	68 (14.8 to 229)	**<0.01**
ΔeGFR 3 m (mL/min/1.73 m^2^), median (IQR)	−1.9 (−7.1 to 3.4)	−6.3 (−11.4 to 0.0)	1.6 (−2.8 to 5.6)	**<0.01**
ΔeGFR 6 m (mL/min/1.73 m^2^), (Median [IQR])	−3.9 (−8.7 to 2.2)	−7.2 (−14.4 to −5.1)	0.5 (−1.6 to 2.5)	**<0.01**
ΔeGFR 12 m (mL/min/1.73 m^2^), (Median [IQR])	−6.3 (−12.4 to −0.4)	−8.2 (−15.7 to −6.1)	−1.8 (−8.4 to 8.4)	**<0.01**
ΔeGFR 24 m (mL/min/1.73 m^2^), (Median [IQR])	−9.3 (−16.6 - 1.9)	−13.1 (−25.3 to −7.5)	0.6 (−11.8 to 6.7)	**<0.01**
Graft loss after 24 m, n (%)	14 (30.1)	12 (52.17)	2 (8.70)	**<0.01**

Abbreviations: AMR, Antibody-mediated Rejection; BMI, Body Mass Index; BKPyVAN, BK Polyomavirus-Associated Nephropathy; m, months; eGFR, CKD-EPI-estimated glomerular filtration rate; HLA, Human Leukocyte Antigen; IQR, interquartile range; mL, milliliter; MRI, Magnetic Resonance Imaging; TCMR, T-cell-mediated Rejection; TMA, Thrombotic microangiopathy.

Bold values indicate significant differences.

### Biopsy Findings

Thirty-seven biopsies (80.4%) were performed based on clinical indications, primarily due to the deterioration of graft function, while the other nine biopsies (19.6%) were protocol biopsies. In 14 (30.4%) biopsies, graft rejection was diagnosed (see Table 1). The overall rate of rejections was equally distributed between the T_1_^high^ and T_1_^low^ groups (30.4% each, p > 0.99). Antibody-mediated rejection (AMR) was numerically but not significantly higher in the T_1_^high^ group (26.1% vs. 13%, p = 0.45). The T cell-mediated rejection (TCMR) frequency also did not differ significantly between both groups (4.3% vs. 17.4%, p = 0.34). Twenty-six allografts (56.6%) exhibited high-grade interstitial fibrosis (ci 2 or 3), and in 18 kidneys (39.1%), high-grade tubular atrophy (ct 2 or 3) was found (Supplementary Table S1). Allografts in the T_1_^high^ group had more severe interstitial fibrosis: 47.8% with ci 3 compared to 21.7% in the T_1_^low^ group (p = 0.044). Tubular atrophy was also more advanced in the T_1_^high^ group (ct 3: 30.4% versus 8.7% in the T_1_^low^ group, p = 0.031). Although not statistically significant, arterial intimal thickening showed higher severity in the T_1_^high^ group (52.2% at cv 2 compared to 34.8% in the T_1_^low^ group, p = 0.059). The severity of glomerular basement membrane double contours (cg), did not differ between the groups; cg grades 2 or 3: 22.7% in the T_1_^high^ group vs. 14.2% in the T_1_^low^ group (p = 0.14). Chronicity index differed significantly between the groups: T_1_^high^ 8.5 (5–11) vs. 3 (IQR 2.5–6.5) in the T_1_^low^ group, p < 0.01.

### Correlation of T_1_ With Histology and Baseline Variables

**FIGURE 1:**
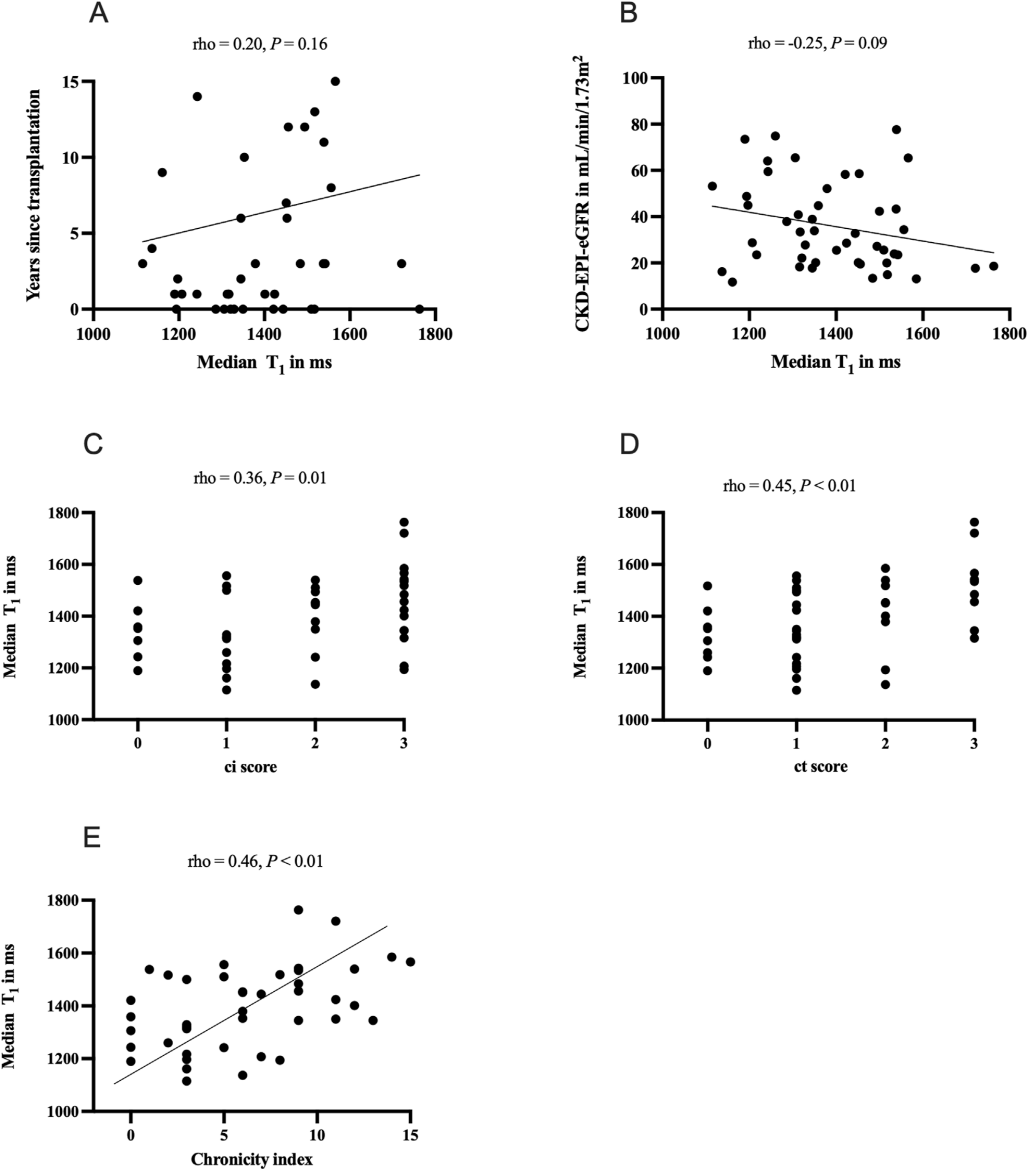
Correlations of clinical and histological parameters and T_1_ relaxation times: panel **(A)** correlation of time since transplantation and median T_1_ in ms; panel **(B)** correlation of baseline estimated glomerular filtration rate (CKD-EPI-eGFR) and median T_1_ in ms; panel **(C)** correlation of interstitial fibrosis (Banff ci score) and median T_1_ in ms; panel **(D)** correlation of tubular atrophy (Banff ct score) and median T_1_ in ms, panel **(E)** correlation of chronicity index and T_1_ median in ms. The chronicity index described by Haas et al. [24] combines interstitial fibrosis (ci), tubular atrophy (ct), vascular fibrous intimal thickening (cv), and chronic glomerulopathy (cg).

There was a significant positive correlation between median T_1_ and interstitial fibrosis (ρ = 0.36, p = 0.01) as well as tubular atrophy (ρ = 0.45, p < 0.01). Further on, the chronicity index correlated positively with T_1_ (ρ = 0.46, p < 0.01). No significant correlation was found between median T_1_ and the time since transplantation (ρ = 0.20, p = 0.16). T_1_ did not correlate with median eGFR at baseline (ρ = −0.25, p = 0.09, see Figure 1).

### Analysis of Graft Function in Relation to T_1_

**FIGURE 2:**
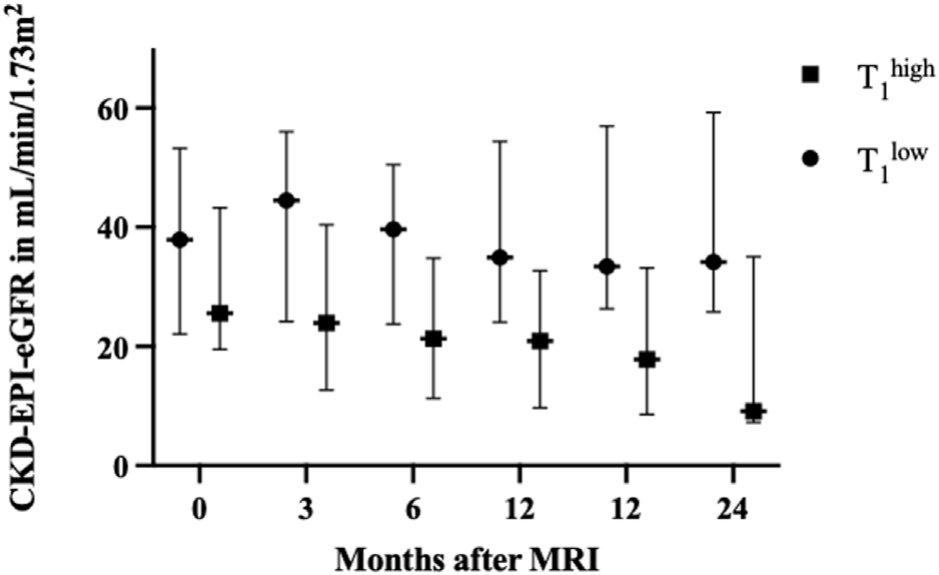
Renal graft function during the follow-up period, compared between the T_1_^high^ and T_1_^low^ groups. No differences were observed at baseline. By 3 months, the T_1_^high^ group’s median estimated glomerular filtration rate (eGFR) was 23.9 (12.7–40.4) mL/min/1.73 m^2^, compared to 44.5 (24.2–56.01) mL/min/1.73 m^2^ in the T_1_^low^ group (p = 0.011). At 6 months, the T_1_^high^ group’s median eGFR was 21.34 (11.3–34.8) mL/min/1.73 m^2^ compared to 39.6 (23.8–50.5) mL/min/1.73 m^2^ in the T_1_^low^ group (p = 0.007). This trend continued, with the T_1_^high^ group having a significantly lower median eGFR at 9 months (20.9 [9.7–32.7] mL/min/1.73 m^2^) than the T_1_^low^ group (34.9 [24.1–54.4] mL/min/1.73 m^2^, p = 0.007). By 12 months, the T_1_^high^ group’s median eGFR had decreased to 17.8 [8.6–33.1] mL/min/1.73 m^2^, compared to 33.4 [26.3–56.9] mL/min/1.73 m^2^ in the T_1_^low^ group (p = 0.006). This significant decline persisted at 24 months, where the T_1_^high^ group had a median eGFR of 9.1 (7.3–35.0) mL/min/1.73 m^2^, whereas the T_1_^low^ group maintained a median of 34.1 (25.8–59.2) mL/min/1.73 m^2^ (p = 0.005). Values of eGFR are shown as median with whiskers indicating the interquartile range. Abbreviations: MRI: magnetic resonance imaging, CKD-EPI-eGFR: estimated glomerular filtration rate calculated with CKD-EPI equation, in mL/min/1.73 m^2^.

In the T_1_^high^ group, eGFR levels consistently declined over time. At baseline, the T_1_^high^ group had a median eGFR of 25.6 [19.6–43.3 (median, IQR)], compared to 37.9 (22.1–53.2) mL/min/1.73 m^2^ in the T_1_^low^ group (p = 0.21) in the T_1_^low^ group (p = 0.20). Across all other time points, the T_1_^high^ group experienced a significant and steady decrease in eGFR (Figure 2). The ΔeGFR between various time points (0–3, 0–6, 0–12, and 0–24 months) indicated a significant decline in graft function in the T_1_^high^ group over all time points. At 3 months, the ΔeGFR was −6.3 (−11.4 to 0.0) mL/min/1.73 m^2^ in the T_1_^high^ group and 1.6 (−2.84 to 5.62) mL/min/1.73 m^2^ in the T_1_^low^ group (p < 0.01). At 24 months, the T_1_^high^ group had a ΔeGFR of −13.0 (−25.3 to −7.48) mL/min/1.73 m^2^ compared to the T_1_^low^ group with 0.6 (−11.80 to 6.68) mL/min/1.73 m^2^ (p < 0.01, see Table 1).

### Correlation of Graft Function and T_1_

We analyzed the correlation between median T_1_ and eGFR values over time. A significant inverse relationship was found between T_1_ and eGFR at different time points. At 3 months, the correlation between T_1_ and eGFR was moderate (ρ = −0.42, p < 0.01). This negative correlation continued at 6 months (ρ = −0.38, p < 0.01), 12 months (ρ = −0.43, p < 0.01), and remained stable at 24 months (ρ = −0.41, p < 0.01). Fisher’s Z transformation analysis between T_1_ and ci association with graft function revealed no significant differences, showing that T_1_ is similarly correlated with kidney function as the established ci score (details see Supplementary Table S2). In the subgroup, including only patients who underwent protocol biopsies, we also found significant correlations between T_1_ and eGFR at months 3 (ρ = −0.71, p = 0.047), 9 (ρ = −0.81, p = 0.015), 15 (ρ = −0.81, p = 0.015), 18 (ρ = −0.74, p = 0.037), 21 (ρ = −0.81, p = 0.015), and 24 (ρ = −0.83, p = 0.010) (see Supplementary Tables S3, S4 for details).

### eGFR Slope

**FIGURE 3:**
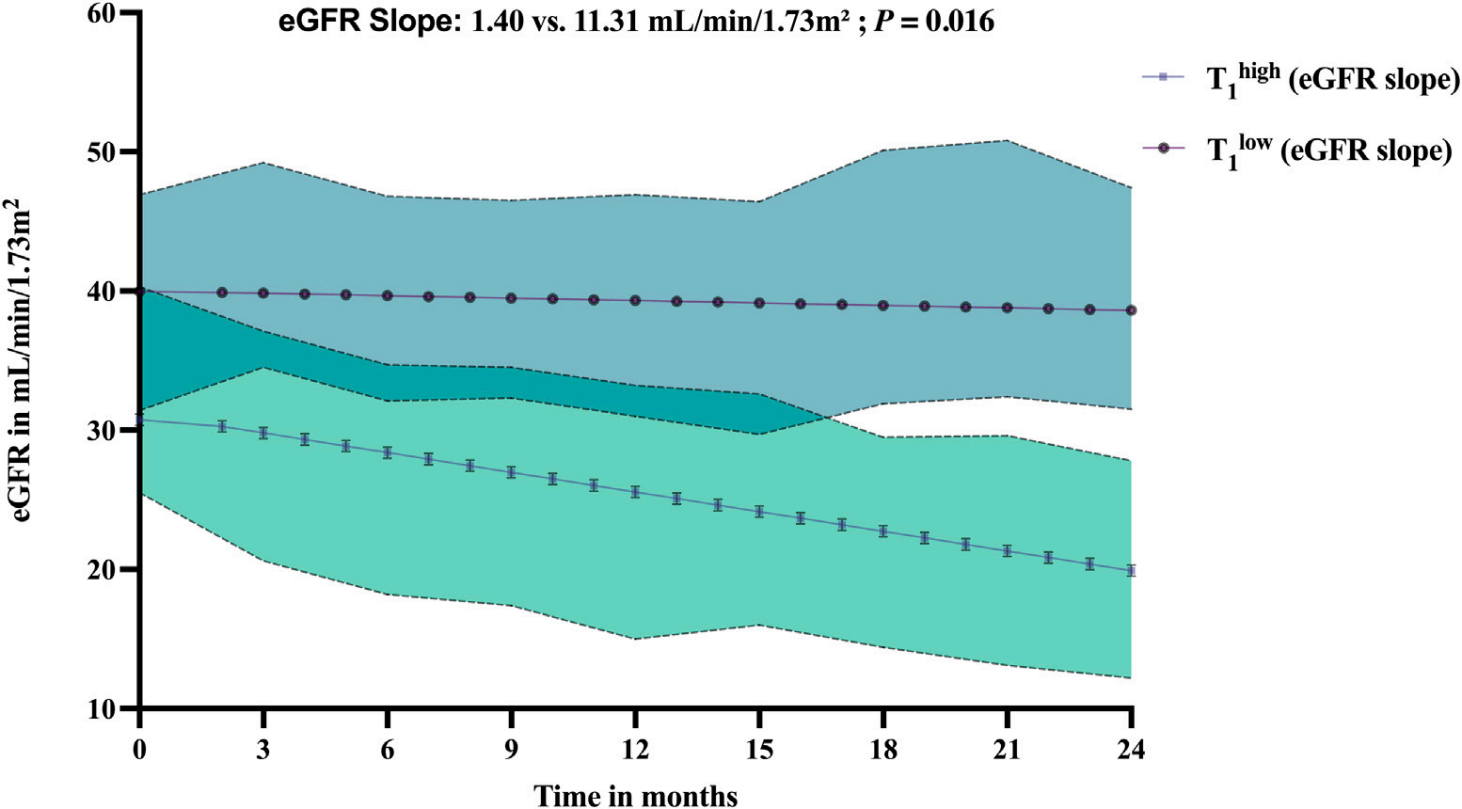
Comparative analysis of estimated glomerular filtration rate (eGFR) values (presented with 95% confidence intervals as shaded areas) over 24 months in kidney transplant recipients compared between the T_1_^high^ and T_1_^low^ groups. Abbreviations: eGFR, estimated glomerular filtration rate calculated with CKD-EPI equation, in mL/min/1.73 m^2^.

The baseline (month 0) eGFR intercept for the T_1_^low^ cohort was 39.9 mL/min/1.73 m^2^, while the T_1_^high^ group had a baseline eGFR intercept that was 9.20 units lower (p = 0.096). Over time, the T_1_^low^ group showed a slight, non-significant decline in eGFR at a rate of 0.06 mL/min/1.73 m^2^ per month (p = 0.63). In contrast, the T_1_^high^ group experienced a significantly steeper decline, with an additional 0.41 units per month (p = 0.016) compared to the T_1_^low^ group. This resulted in a total eGFR decline of 11.31 mL/min/1.73 m^2^ for the T_1_^high^ group and 1.40 mL/min/1.73 m^2^ for the T_1_^low^ group over 24 months (Figure 3).

### ROC Analysis

**FIGURE 4:**
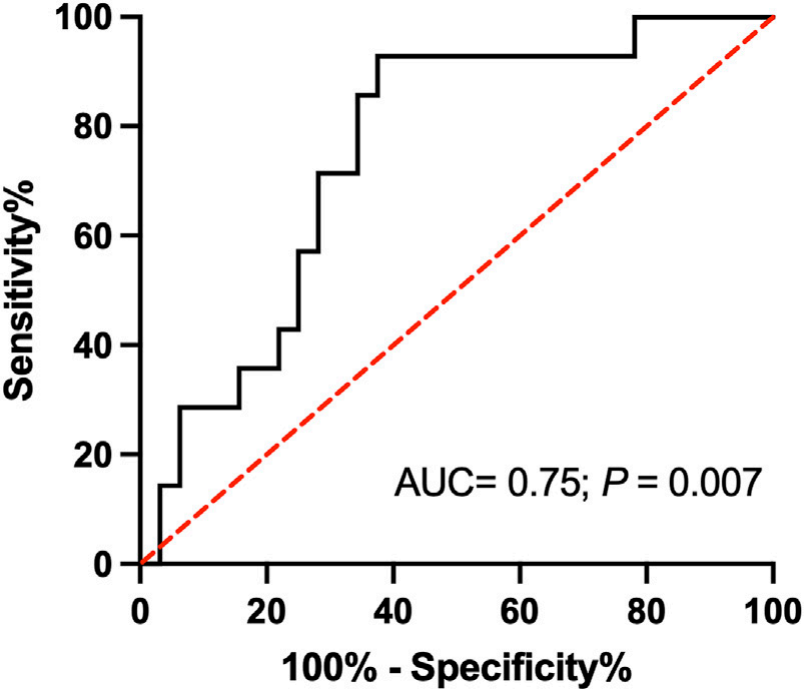
Receiver operating characteristic (ROC) analysis for T_1_ relaxation time and graft loss after 24 months of follow-up. Higher T_1_ values indicate a higher likelihood of graft loss. The optimal cutoff of “> 1,352 ms” provides the best balance for accurately identifying patients with an increased risk of graft loss. Abbreviation: AUC, area under the curve, ms: millisecond.

We used ROC analysis to assess if T_1_ can be used as a predictive marker for renal allograft loss (Figure 4). T_1_ above the median resulted in a PPV for predicting graft loss of 52.2% with an AUC of 0.75, p = 0.007. Conversely, the NPV was 91.3%. T_1_ demonstrated a sensitivity of 100% across the lower cutoff values, specifically from “>1,126 ms” to “>1,317 ms”. At the cutoff of “>1,317 ms”, the sensitivity slightly decreased to 92.9%, while the specificity saw a substantial increase, indicative of fewer false-positive results. At “>1,337 ms”, sensitivity is still 92.86%, but specificity has increased to 53.1%. At “>1,352 ms”, the sensitivity remained at 92.86%, and the specificity increased further to 62.5%. The analysis identifies T_1_ “>1,352” ms as an optimal cutoff point in our patient cohort for balancing sensitivity and specificity in a clinical setting.

### Survival Analysis and Kaplan-Meier Curve

**FIGURE 5:**
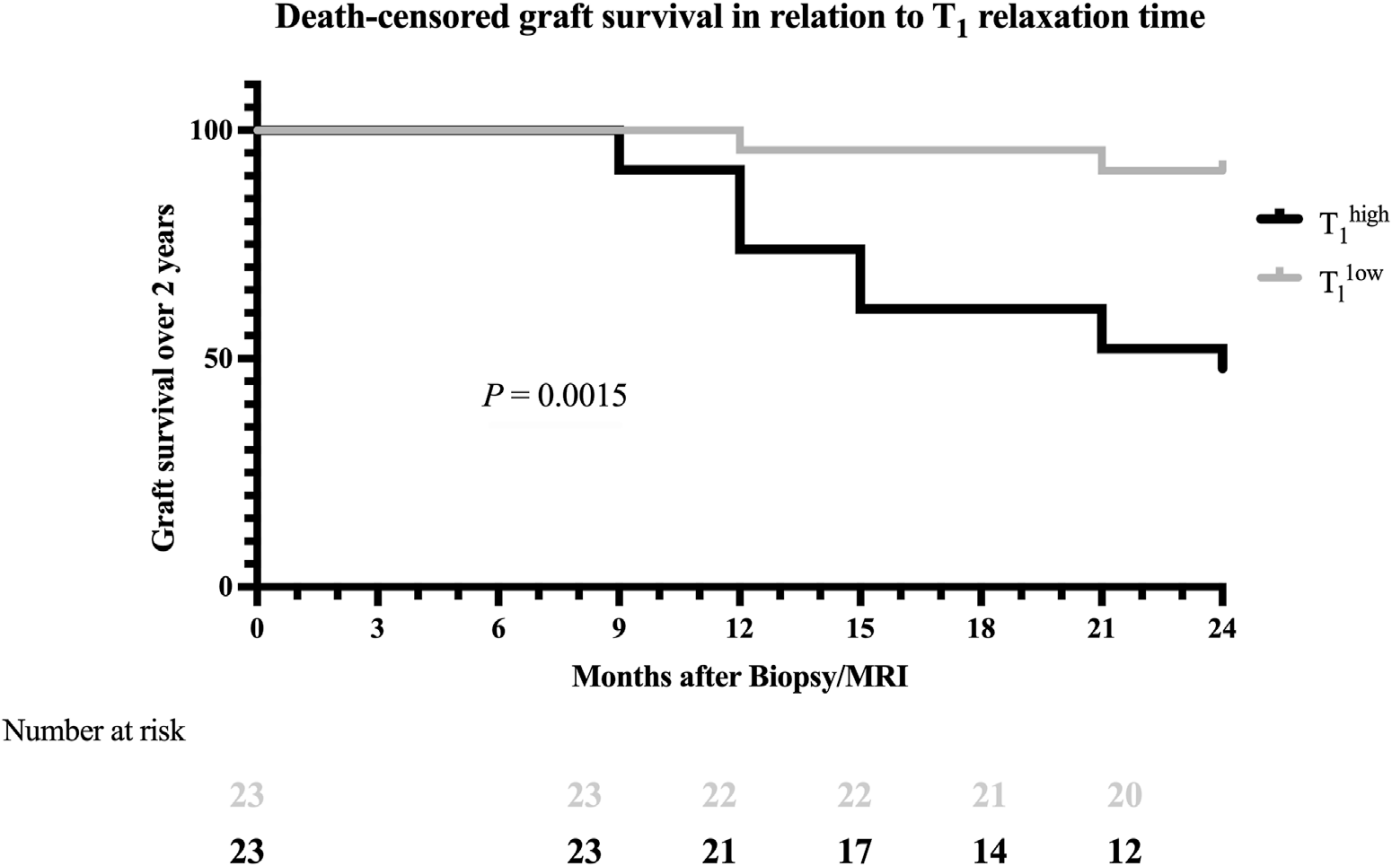
Kaplan-Meier survival curve for the death-censored graft survival analysis 24 months after study enrollment. The black line illustrates graft survival for T_1_^high^ patients, and the grey line illustrates graft survival for T_1_^low^ patients. Within the timeframe of 24 months, a significant difference in graft survival was found (Log-rank test: p = 0.0015).

The Kaplan-Meier survival analysis revealed significant differences in graft survival between the groups (Figure 5). After 12 months, all kidney transplants in the T_1_^low^ group were still functioning, compared to 91.3% in the T_1_^high^ group. This difference became more pronounced over time, with survival rates of 91.3% versus 60.9% at 21 months and 87.0% versus 52.2% after 24 months (Log-rank test, p = 0.0015, Figure 5). A T_1_ above the median was a significant risk factor for graft loss (HR 7.3, 95% CI: 2.6–21.0). The cortico-medullary difference of the T_1_ (ΔT_1_) was available in 32 patients. Patients without graft loss had a mean ΔT_1_ of −337.13 ms, while those with graft loss had a mean of −251.81 ms, with no significant differences (p = 0.417).

## Discussion

We had hypothesized that T_1_, as measured by MRI, could serve as a reliable non-invasive biomarker for predicting kidney allograft dysfunction. T_1_ mapping is an emerging tool to quantify high-grade interstitial fibrosis in renal allografts [11, 15, 25]. Yet, little is known about the prognostic relevance of T_1_, a prerequisite for broader use as a non-invasive surveillance tool.

As a major finding of our study, we were able to show that elevated cortical T_1_ not only correlates with histological markers for chronic lesions but can also predict worsening allograft function. Patients with T_1_ above the median had eGFR levels comparable to the T_1_^low^ group at baseline but significantly worse graft function across all follow-up intervals. We further compared the predictive power with established markers of chronic allograft injury, such as interstitial fibrosis. The Z scores, ranging from −0.08 to 1.03, indicate that the correlation of T_1_ with eGFR levels is slightly lower than that of Banff ci across all time points. Yet, the magnitude of the Z scores suggests that these differences are small and not significant, highlighting the potential utility of T_1_ mapping as an accurate, non-invasive alternative to quantify chronic allograft injuries.

Similar results were previously published by Bane et al., where, as part of a multiparametric MRI, T_1_ and diffusion-weighted imaging (cortical ADC values) allowed good prediction of eGFR decline after 18 months [17]. Yet, in comparison to our study, only 12 patients with allograft dysfunction underwent biopsies, and those were performed at more variable time intervals. With the higher sample size and a longer follow-up period of our study, we were not only able to confirm the findings from Bane et al. but showed that also cortical T_1_ alone allows a decent prediction of graft function during midterm follow-ups. As the measurement of cortical T_1_ times alone is less time-demanding as a multiparametric protocol, it may further facilitate the implementation of MRI in post-transplant surveillance programs.

A previous study from Shi et al. reported that cortical T_1_ was associated with higher fibrosis and worse renal outcomes in native kidneys [26]. Interestingly, similar to the study from Shi et al., we observed that in some patients with the lowest Banff ci score (ci 0), cortical T_1_ was above our median split value. Whether this was due to sampling error in the biopsy or based on other factors influencing MRI results remains speculative [15, 25, 27]. In a previous study from Berchtold et al., it was shown that altered T_1_ might even precede the development of histological signs of chronic injury [28]. Besides chronic fibrosis, animal studies with ischemia-induced acute kidney injury showed that T_1_ also correlates with the degree of capillary leakage and both cellular and interstitial edema, essential components of acute local inflammation. Unfortunately, our subgroup of patients with ci 0 was too small to study this finding in more detail.

Moreover, our research gave insight into the prognostic implications of T_1_ through ROC analysis and Kaplan-Meier survival curves. The high NPV of T_1_ suggests that magneto-chemical alterations caused by morphological changes associated with deterioration of graft function are absent, and probability of short-term graft loss is low. Concurrently, the Kaplan-Meier analysis demonstrated a significant survival advantage for allografts with lower T_1_, further cementing the potential prognostic relevance of renal MRI in post-transplant care. A new aspect of our study was the exploration of eGFR slopes over time, the currently most endorsed method to quantify renal function declines [29].

Certain limitations in our study need to be addressed. We focused our analysis on T_1_ and did not include other MRI methods. On the other hand, we were able to show that even with one single MRI parameter, meaningful prognostic estimates are possible. The study’s sample size, while adequate for preliminary analysis, necessitates larger, multicenter trials to validate our findings across diverse populations and clinical settings. The use of the last observation carried forward (LOCF) method to address data discontinuity due to graft loss, while methodologically sound, may introduce a conservative bias, potentially underestimating the predictive power of T_1_. Additionally, the study’s reliance on a single MRI parameter, despite its advantageous application capabilities, might not capture the entirety of the post-transplant complexities. It is also noteworthy that a number of patients in the T_1_^high^ group were diagnosed with antibody-mediated rejections in their biopsies, possibly indicating a more aggressive underlying disease. Whereas in the T_1_^low^ group, pathologies with potentially benign outcomes such as BKPyVAN were found, our MRIs were performed between 2017 and 2019, a time before the emerging AMR treatments were available [30]. Results from our ROC analysis are based on a relatively high graft loss rate, especially in the T_1_^high^ in our patient population. Yet, to apply our reported PPV and NPV values in an overall renal transplant cohort, further studies including more stable renal grafts (e.g., only protocol biopsies) may be necessary.

In conclusion, our study contributes to the growing field of renal transplant diagnostics by highlighting the prognostic value of T_1_. Yet, the adoption of MRI in routine post-transplant monitoring still hinges on considerations of cost, accessibility, and the standardization of imaging protocols [11, 31]. By demonstrating the potential to identify patients at high risk for midterm graft failure, we further add to the growing data, highlighting the potential utility of this non-invasive marker. Future research, encompassing larger cohorts and longitudinal studies, will be instrumental in integrating MRI into kidney transplant surveillance.

## Data Availability

The raw data supporting the conclusions of this article will be made available by the authors, upon reasonable request.

## Abbreviations

AMR, antibody-mediated rejection; ADC, apparent diffusion coefficient; BKPyVAN, BK Polyomavirus associated nephropathy; ci, Banff score for interstitial fibrosis; ct, Banff score for tubular atrophy; cg, Banff score for double contours; cv, Banff score for vascular fibrous intimal thickening; DWI, diffusion-weighted imaging; HR, hazard ratio; GFR, glomerular filtration rate; IQR, interquartile range; LOCF, last observation carried forward; mo/mos, month/months; MRI, magnetic resonance imaging; ms, milliseconds; NPV, negative predictive value; PPV, positive predictive value; SD, standard deviation; TCMR, T cell-mediated rejection; TMA, thrombotic microangiopathy; Tx, transplantation.
