# Supplementary material for: T1 Relaxation Time for the Prediction of Renal Transplant Dysfunction
Source: Transpl Int. 2025 Apr 10;38:14301. doi: 10.3389/ti.2025.14301 (PMC12018245; doi:10.3389/ti.2025.14301)
Supplement: Supplementary file 1 [file DataSheet1.pdf]

Supplemental Table S1.

Histological Lesion	T_1^{high}	T_1^{Low}	Overall	P-Value
**g**				0.53
- .00	13 (59.1%)	16 (76.2%)	29 (67.4%)	
- 1.00	4 (18.2%)	3 (14.3%)	7 (16.3%)	
- 2.00	1 (4.5%)	1 (4.8%)	2 (4.7%)	
- 3.00	4 (18.2%)	1 (4.8%)	5 (11.6%)	
**i**				0.55
- .00	17 (77.3%)	17 (81.0%)	34 (79.1%)	
- 1.00	4 (18.2%)	3 (14.3%)	7 (16.3%)	
- 2.00	0 (0.0%)	1 (4.8%)	1 (2.3%)	
- 3.00	1 (4.5%)	0 (0.0%)	1 (2.3%)	
**ti**				0.12
- .00	7 (30.4%)	11 (50.0%)	18 (40.0%)	
- 1.00	8 (34.8%)	4 (18.2%)	12 (26.7%)	
- 2.00	3 (13.0%)	6 (27.3%)	9 (20.0%)	
- 3.00	5 (21.7%)	1 (4.5%)	6 (13.3%)	
**t**				0.78
- .00	16 (69.6%)	15 (71.4%)	31 (70.5%)	
- 1.00	3 (13.0%)	1 (4.8%)	4 (9.1%)	
- 2.00	3 (13.0%)	4 (19.0%)	7 (15.9%)	
- 3.00	1 (4.3%)	1 (4.8%)	2 (4.5%)	
**v**				0.32
- .00	20 (95.2%)	20 (100.0%)	40 (97.6%)	
- 1.00	1 (4.8%)	0 (0.0%)	1 (2.4%)	
**ah**				0.16
- .00	7 (30.4%)	10 (47.6%)	17 (38.6%)	
- 1.00	7 (30.4%)	1 (4.8%)	8 (18.2%)	
- 2.00	2 (8.7%)	3 (14.3%)	5 (11.4%)	
- 3.00	7 (30.4%)	7 (33.3%)	14 (31.8%)	
**cg**				0.14
- .00	14 (63.6%)	18 (85.7%)	32 (74.4%)	
- 1.00	3 (13.6%)	0 (0.0%)	3 (7.0%)	
- 2.00	2 (9.1%)	0 (0.0%)	2 (4.7%)	
- 3.00	3 (13.6%)	3 (14.3%)	6 (14.0%)	
**ci**				**0.04**
- .00	2 (8.7%)	5 (22.7%)	7 (15.6%)	
- 1.00	3 (13.0%)	9 (40.9%)	12 (26.7%)	
- 2.00	7 (30.4%)	3 (13.6%)	10 (22.2%)	
- 3.00	11 (47.8%)	5 (22.7%)	16 (35.6%)	
**ct**				**0.03**
- .00	2 (8.7%)	6 (27.3%)	8 (17.8%)	
- 1.00	7 (30.4%)	12 (54.5%)	19 (42.2%)	
- 2.00	7 (30.4%)	2 (9.1%)	9 (20.0%)	
- 3.00	7 (30.4%)	2 (9.1%)	9 (20.0%)	
**cv**				0.06
- .00	2 (9.5%)	7 (41.2%)	9 (23.7%)	
- 1.00	3 (14.3%)	2 (11.8%)	5 (13.2%)	
- 2.00	12 (57.1%)	8 (47.1%)	20 (52.6%)	
- 3.00	4 (19.0%)	0 (0.0%)	4 (10.5%)	
**Chronicity Index**				
Median, IQR	8.5 (5 – 11)	3 (2.5- 6.5)	6 (3 – 9)	<**0.01**

Supplemental Table S1.
Histological lesions in the kidney transplant biopsy according to BANFF criteria and group differences. Abbreviations: g: Glomerulitis, i: Interstitial inflammation, ti: Total inflammation, t: Tubulitis, v: Intimal arteritis, ah: Arteriolar hyalinosis, cg: Double contours (glomerular basement membrane duplication), ci: Interstitial fibrosis, ct: Tubular atrophy, cv: Intimal thickening

Supplemental Table S2.

Month	Z Score	P-value
0	1.03	0.30
3	0.51	0.61
6	0.62	0.54
9	0.46	0.65
12	-0.08	0.95
15	-0.04	0.97
18	-0.12	0.91
21	0.11	0.92
24	0.09	0.91

Supplemental Table S2.

The table illustrates the comparison of correlation coefficients between T_1 relaxation times and estimated glomerular filtration rate (eGFR), Banff ci, and eGFR over various time points, using Fisher's Z transformation. The Z-values measure the difference in the strength of correlation between the two sets of correlations. None of the Z-values demonstrate a statistically significant difference, as all associated p-values are above the threshold of 0.05. This indicates no significant variance in how T_1 relaxation times and ci correlate with eGFR over time. The results suggest that both T_1 and ci are comparably related to the changes in kidney function as measured by eGFR across the studied intervals.

Supplemental Table S3.

Month	Z Score	P-value
0	-1.22	0.22
3	-0.95	0.34
6	-1.00	0.31
9	-0.86	0.39
12	-0.03	0.97
15	-0.04	0.97
18	-0.02	0.98
21	-0.40	0.69
24	-0.32	0.75

Supplemental Table S3.

The table presents the results of a subanalysis conducted on a group of patients with protocol biopsies. It shows the Z-difference values and corresponding p-values for comparing the Fisher Z-transformations of correlations between two variables, Banff ci, and eGFR, over various time points. The Z-difference quantifies the difference between the two sets of correlations, while the p-values indicate the statistical significance of these differences. All p-values are above 0.05, indicating that the differences in correlations between T_1 and ci are not statistically significant at any time point in this subanalysis group.

Supplemental Table S4.

Month	Z Score	P-value
0	0.06	0.95
3	-0.27	0.79
6	-0.31	0.76
9	-0.36	0.72
12	0.4	0.69
15	-0.36	0.72
18	0.74	0.46
21	-0.36	0.72
24	-0.14	0.89

Supplemental Table S4.
This table presents the Z-difference values and their corresponding p-values for the comparison between the Fisher Z-transformations of correlations from $\mathbf{T_1}$ and Banff **ci** in a subgroup of patients with indication biopsies. The p-values indicate that the differences between the two variables' correlations are not statistically significant at any time point, as all p-values exceed 0.05.
